# Influence of accessory sulci of the frontoparietal operculum on gray matter quantification

**DOI:** 10.3389/fnana.2022.1022758

**Published:** 2023-01-19

**Authors:** Mariana N. Vallejo-Azar, Lucia Alba-Ferrara, Arabella Bouzigues, Juan P. Princich, Martin Markov, Mariana Bendersky, Paula N. Gonzalez

**Affiliations:** ^1^Unidad de Estudios en Neurociencias y Sistemas Complejos, CONICET, Hospital El Cruce Dr, “Néstor C. Kirchner”, Universidad Arturo Jauretche, Buenos Aires, Argentina; ^2^INSERM U1127, Institut du cerveau, Sorbonne Université, Hôpital Pitié-Salpêtrière, Paris, France; ^3^Laboratorio de Anatomía Viviente, Facultad de Medicina, Universidad de Buenos Aires, Ciudad Autónoma de Buenos Aires, Buenos Aires, Argentina

**Keywords:** frontal operculum, parietal operculum, diagonal sulcus, triangular sulcus, accessory sulci, destrieux atlas

## Abstract

**Introduction:** The perisylvian region is the cortical core of language and speech. Several accessory sulci have been described in this area, whose presence could modify the results of the automatic quantification of gray matter by popularly used software. This study aimed to assess the expression of accessory sulci in the frontoparietal operculum (FPO) and to evaluate their influence on the gray matter volume estimated by an automatic parcellation of cortical gyri and sulci.

**Methods:** Brain MRI scans of 100 healthy adult volunteers were visually analyzed. The existence of the triangular and diagonal sulci, and the number of accessory sulci in the frontoparietal operculum, were assessed on T1 images. Also, the gray matter volume of gyri and sulci was quantified by an automatized parcellation method. Interhemispheric differences in accessory sulci were evaluated with Chi-square and Wilcoxon paired tests. The effects of the hemisphere, sex, age, total intracranial volume, and accessory sulci on morphometric variables were assessed by linear models.

**Results:** These sulci were found in more than half of the subjects, mostly in the left hemisphere, and showed a significant effect on the gray matter content of the FPO. In particular, the volume of the inferior frontal sulcus, pars opercularis of the inferior frontal gyrus, horizontal ramus of the lateral sulcus, angular gyrus, and postcentral gyrus showed a significant influence on the presence of accessory sulci.

**Discussion:** The prevalence of tertiary sulci in the FPO is high, although their meaning is not yet known. Therefore, they should be considered to reduce the risk of misclassifications of normal variation.

## Introduction

**Figure 1:**
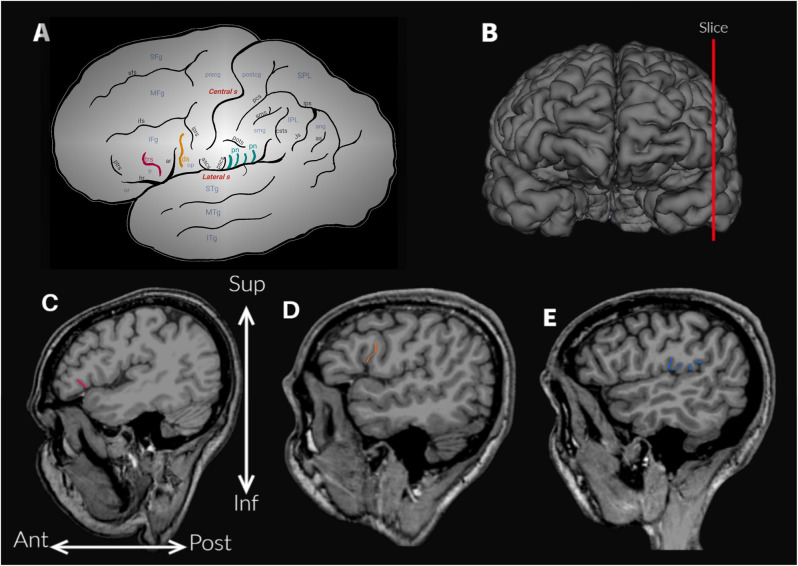
**(A)** Schematic outline of the lateral surface of the hemisphere to illustrate the sulci and gyri of the frontoparietal operculum. Abbreviations: ar, ascending anterior ramus of the lateral sulcus; ang, angular gyrus; ans, angular sulcus; ascs, anterior subcentral sulcus; csts, caudal superior temporal sulcus; ds, diagonal sulcus (drawn in orange); hr, horizontal ramus of the lateral sulcus; IFg, inferior frontal gyrus; ifs, inferior frontal sulcus; IPL, inferior parietal lobule; iprs, inferior precentral sulcus; ips, intraparietal sulcus; ITg, inferior temporal gyrus; Js, Jensen sulcus; MFg, middle frontal gyrus; MTg, middle temporal gyrus; op, opercular part (or pars opercularis) of the inferior frontal gyrus; or, orbital part (or pars orbitalis) of the inferior frontal gyrus; pn, parietal notches of the lateral sulcus (drawn in light blue); pcs, postcentral sulcus; postg, postcentral gyrus; pots, postcentral transverse sulcus; precg, precentral gyrus; prts, pretriangular sulcus; pscs, posterior subcentral sulcus; SFg, superior frontal gyrus; sfs, superior frontal sulcus; smg, supramarginal gyrus; sms, supramarginal sulcus; SPL, superior parietal lobule; sps, superior parietal sulcus; STg, superior temporal gyrus; tr, triangular part (or pars triangularis) of the inferior frontal gyrus; trs, triangular sulcus (drawn in pink). **(B)** Approximate location of the parasagittal slices analyzed to assess the accessory sulci. **(C)** Triangular sulcus. **(D)** Diagonal sulcus. **(E)** Parietal notches.

The frontoparietal operculum (FPO) comprises regions of the frontal and parietal cortex, dorsal to the lateral sulcus (Sylvian fissure), including the inferior frontal gyrus and the ventral parts of the precentral and postcentral gyri, subcentral gyrus, supramarginal and angular gyri (Petrides, 2013; Figure 1A). As it is well known, the perisylvian region is the cortical core of language (Knaus et al., 2006; Petrides, 2013). It has been extensively studied, from the first postmortem brain analyses of aphasic patients performed in the 19th century, to current studies based on neuroimaging techniques (Petrides, 2013; Tremblay and Dick, 2016). The anatomical boundaries of some of the regions of the FPO are not clearly defined (Seghier, 2013; Tremblay and Dick, 2016), and the use of different criteria results in high variability among studies about their morphometric and morphologic characteristics (Keller et al., 2009; Amunts and Zilles, 2012; Tremblay and Dick, 2016). The angular and supramarginal gyri illustrate this situation, given that different landmarks (i.e., sulci) and imaginary lines have been proposed to delimit them from neighboring lobes (Petrides, 2013). For example, in the automatic parcellations of the human cortex -such as the work by Destrieux et al. (2010), the angular gyrus is delimited by two sulci (i.e., intraparietal sulcus and sulcus intermedius primus) and also two virtual lines (a temporoparietal line and a parietooccipital line).

A less studied variation in structural analyses involving areas of the FPO is the interindividual variability in the expression of secondary and tertiary sulci, usually called “accessory sulci” (Ono et al., 1990; Petrides, 2013). Accessory or tertiary sulci emerge late in gestation, and many have evolutionary relevance (Miller and Weiner, 2022). These sulci are usually small and shallow and exhibit high morphological individual variability (Miller and Weiner, 2022). This variability is particularly relevant for the FPO because different accessory sulci have been found in variable numbers, and their expression can differ between hemispheres (Ono et al., 1990; Keller et al., 2007; Petrides, 2013; Idowu et al., 2014; Eser Ocak and Kocaelı, 2017). The anatomical variation of the perisylvian region has been studied especially in the temporal and frontal operculum (e.g., Ochiai et al., 2004; Knaus et al., 2006; Keller et al., 2007, 2009; Idowu et al., 2014), particularly in areas classically known as Broca’s and Wernicke’s areas -terms without anatomical consistency today (Tremblay and Dick, 2016). In contrast, the study of the parietal operculum has been relatively overshadowed (Ide et al., 1996; Toga and Thompson, 2003; Seghier, 2013; Eser Ocak and Kocaelı, 2017; Palomero-Gallagher and Zilles, 2019). Particularly, variability described in the inferior frontal gyrus includes the presence of the triangular and diagonal accessory sulci in the pars triangularis and pars opercularis respectively, the pattern of the inferior frontal and inferior precentral sulci, the shape of the pars triangularis, and the morphology of the rami of the lateral sulcus (e.g., Keller et al., 2007; Idowu et al., 2014; Eser Ocak and Kocaelı, 2017; Sprung-Much and Petrides, 2018). Only some focused on the parietal operculum and has analyzed the expression of accessory sulci (Ono et al., 1990) and the morphological patterns of constant sulci such as the central sulcus and anterior and posterior subcentral sulci (Eichert et al., 2021).

The accessory sulci have been overlooked in the field due to methodological obstacles, but recent studies (e.g., Miller et al., 2021; Voorhies et al., 2021; Miller and Weiner, 2022) suggest a relationship between the morphology of these sulci with cortical network and cognition which highlights the relevance of their investigation. In addition, the knowledge about sulcal patterns of FPO is of importance as these sulci could represent anatomical landmarks which could help define with greater precision neurofunctional activity (Petrides, 2013; Eichert et al., 2021), and to establish baselines for studies about folding parameters in diseases or throughout development and evolution. Neurosurgical techniques that expose the lateral sulcus, such as the pterional, or its reduced variant, mini-pterional approaches, require a clear topographical comprehension of cerebral gyri and sulci for the localization of cortical lesions and decisions concerning surgical treatment. Besides, the intraoperative recognition of cortical landmarks enables neurosurgeons to preserve eloquent fields (Ribas, 2010).

The anatomical variability of the FPO can also have important effects on the quantification of cortical gray matter given that the presence of accessory sulci may increase the sulcal tissue to sample (Keller et al., 2009). This can be particularly problematic for the field of quantitative neuroimaging due to the use of computational neuroanatomical atlases to automatically parcel the cortex into regions of interest that are based on constant anatomical traits, a widely used method in clinical practice and research today. Automatic parcellations locate, identify and label cortical gyri and sulci after registration to a template to obtain measures such as volume, surface area, and thickness (Destrieux et al., 2010). In this process, variable features, such as “accessory” or “inconstant” sulci are merged into parcels that correspond to constant sulci to obtain units that can be consistently parcellated (Destrieux et al., 2010). The inclusion of accessory sulci into parcels corresponding to constant sulci can increase the amount of gray matter estimated in neighboring parcels. However, the influence of individual variations on automatic quantification has not been studied in detail.

This work aimed to assess the anatomical inter and intra-individual variability of the FPO and to test the influence of the expression of the accessory sulci on cortical gray matter quantification. In contrast to previous studies, mostly based on small samples *ex vivo* or a reduced number of magnetic resonance images (MRIs), we study a large sample of MRIs from healthy volunteers. We particularly assessed the presence and the number of accessory sulci by a visual survey and estimated the volume of cortical gyri and sulci using an automatic segmentation pipeline implemented in Freesurfer (Fischl et al., 1999).

## Material and Methods

### Sample and MRI acquisition

We recruited 100 healthy volunteers (43 males and 57 females) living in the Buenos Aires Metropolitan Area (Argentina) that met the requirements for inclusion in the sample. These criteria comprised no known neurological or psychiatric disease or systemic disease which could impact neurological function and not taking any medication which could have effects on neurological function. Subjects with implanted metal clips or wires are not allowed to undergo MRI scanning and so were excluded. The mean age was 31.4 years (range: 18–57). Subjects were right-handed, as revealed by the Edinburgh Handedness Inventory (given the small sample size of left-handed and ambidextrous subjects, these were excluded from this study, *n* = 13). All volunteers had at least 12 years of education, 48.96% were undergraduate students, and 36.46% had completed a university qualification. Participants gave their informed consent to participate in the study, approved by the Ethical Committees of the Hospital El Cruce and Hospital Angel Roffo, in agreement with the Declaration of Helsinki ethical standards. All the acquired images were visually inspected by an expert neuroradiologist (JP) to exclude subjects with incidental findings.

Subjects were scanned in two scanners, a 3T Philips Achieva (*n* = 55) located in the Hospital El Cruce in Florencio Varela (second urban belt of the metropolitan area of Buenos Aires) and a 3T Siemens Trio (*n* = 45) located in the Hospital Angel Roffo from Buenos Aires. Volumetric T1 images were obtained using a 3D FFE sequence (TE = 3.3 ms, TR = 2,300 ms, TI = 900 ms, flip angle = 9°, FOV = 240 × 240 × 180, voxel size = 1 × 1 × 1 mm^3^ and 239 slices) in the 3T Philips Achieva, and MP-RAGE sequence (TE = 2.27 ms, TR = 2,000 ms, TI = 900 ms, inverted angle = 80, FOV = 250 × 250, voxel size = 1 × 1 × 1 mm^3^ and 204 slices) in the 3T Siemens Trio. The images were converted from DICOM to NIFTI format using Dcm2Nii^1^.

### Assessment of accessory sulci

**Figure 2:**
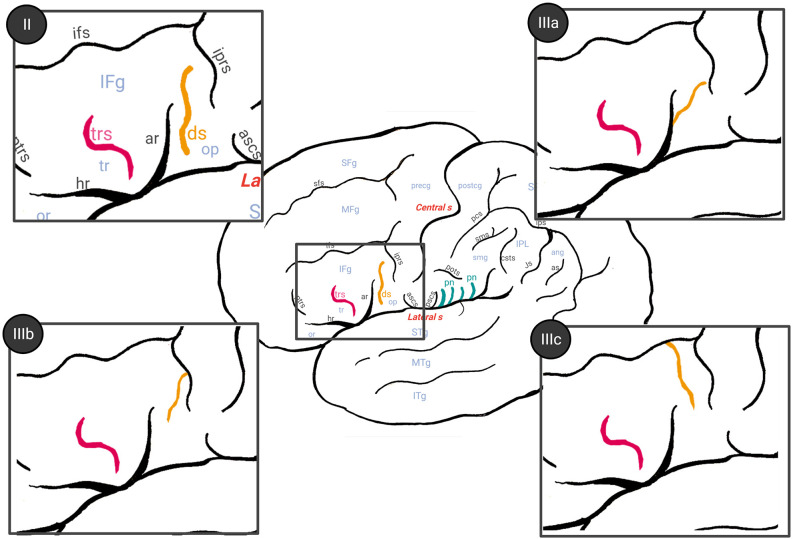
Different patterns of diagonal sulcus previously described by Sprung-Much and Petrides (2018) (drawing based on that article). Color legend: ds (in orange), diagonal sulcus; trs (in pink), triangular sulcus. Type II: diagonal sulcus is separated with respect to other neighboring sulci. Type IIIa: diagonal sulcus joins superficially with the anterior ascendent ramus of the lateral sulcus. Type IIIb: idem but with the inferior precentral sulcus. Type IIIc: diagonal sulcus links superficially with the inferior frontal sulcus. In the present study, we considered Type I as 0 (absent diagonal sulcus), while type II and III a, b and c were considered as 1 (present).

The anatomical variability of the FPO was evaluated by two trained observers (MV-A and AB), who visually identified the accessory sulci (Table 1) in each hemisphere from MRI imported into the software 3D slicer software^2^. The frontal operculum includes the inferior frontal and precentral gyri (Figure 1A). The inferior frontal gyrus lies below the inferior frontal sulcus and is divided into three parts (pars) by the horizontal and ascending anterior rami of the lateral sulcus. The pars orbitalis is anteroinferior to the horizontal ramus and is medially and inferiorly continued by the orbital gyri (Destrieux et al., 2010). The pars triangularis is located between these two anterior rami of the lateral sulcus (Destrieux et al., 2010). Finally, the pars opercularis is posterior to the ascending anterior ramus and extends to the ventral part of the inferior precentral sulcus (Sprung-Much and Petrides, 2018). In the frontal operculum, we assessed the presence of the triangular and diagonal sulci in each hemisphere (Figures 1A,C,D). The presence or absence of each sulcus was codified as 1 or 0 respectively. When the triangular sulcus is present, it splits the pars triangularis into an anterior and a posterior part (Figure 1C). When present, the diagonal sulcus is a vertically oriented sulcus within the pars opercularis (Figure 1D; Tomaiuolo et al., 1999; Keller et al., 2009; Petrides, 2013; Sprung-Much and Petrides, 2018). Sprung-Much and Petrides (2018) described three main types of morphological patterns of the diagonal sulcus based on if this sulcus is separated or fused with the neighboring sulci (i.e., ascending anterior ramus of the lateral sulcus anteriorly, inferior precentral sulcus posteriorly or inferior frontal sulcus dorsally; Figure 2). Behind the pars opercularis there is a U-shaped convolution that connects the precentral and postcentral gyri, marked by the anterior and posterior subcentral branch of the Sylvian fissure, the subcentral gyrus. However, data about the prevalence of these two sulci are scarce (with exceptions: e.g., Eichert et al., 2021), so we included them in our observations. In the parietal operculum, we identified a set of accessory sulci (besides anterior and posterior subcentral sulci) that interrupt the lateral sulcus in the segment between the posterior ramus and the inferior precentral sulcus (Figures 1A,E). These sulci have only been briefly mentioned by Testut and Latarjet (1981) as branches of the Sylvian fissure that cut the upper lip, the parietal notches, without more specifications. In this study, these sulci (i.e., subcentral sulci and parietal notches) were counted in each hemisphere.

**Table 1:** List of accessory sulci assessed in the present study.

Sulci	Definitions
Triangular sulcus or incisura capitis	This sulcus is split into anterior and posterior parts the pars triangularis of the inferior frontal gyrus (Figure 1; Petrides, 2013). It can be connected with the inferior frontal sulcus (Keller et al., 2007).
Diagonal sulcus	Sulcus is vertically oriented within the pars opercularis of the inferior frontal gyrus. It can be separated from the neighboring sulci, or superficially joined with a neighboring sulcus (Figures 1, 2; Tomaiuolo et al., 1999; Keller et al., 2009; Petrides, 2013; Sprung-Much and Petrides, 2018).
Parietal notches	Set of accessory sulci besides the anterior and posterior subcentral sulci interrupt the lateral sulcus in the segment between the posterior ramus and the inferior precentral sulcus. These sulci have only briefly mentioned by Testut and Latarjet (1981) as branches of the Sylvian fissure that cut the upper lip, the parietal notches, without more specifications (Figure 1).

**Figure 3:**
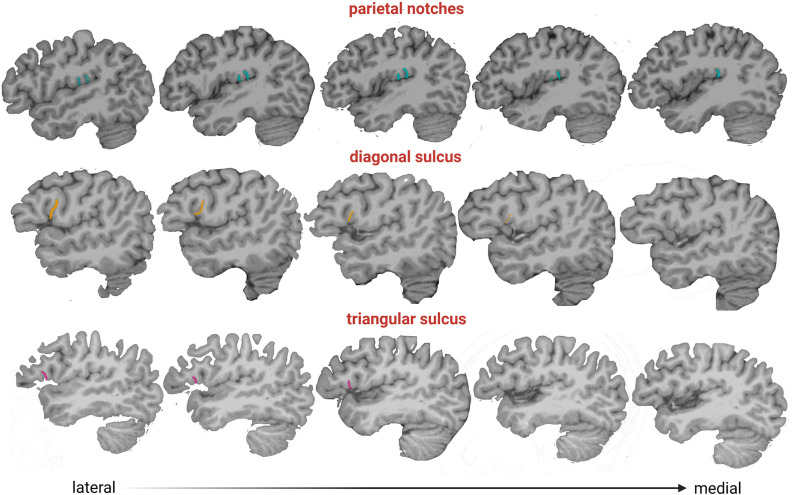
Parasagittal slices from lateral to medial (left to right), where accessory sulci can be observed and their depth. Color legend: pink, triangular sulcus; orange, diagonal sulcus; light blue, parietal notches.

Two observers (MV-A, AB) tested the frequency of the presence of each accessory sulcus on a subset of the sample. The identification of these sulci was carried out until significant differences between observers disappeared. Then, one observer (MV-A) assessed the accessory sulci in the entire sample, and the results were checked by an experienced neuroanatomist (MB). The sulci were visualized in parasagittal MRI sections and verified in orthogonal slices (Figures 1B–E), navigating the slices from the lateral surface towards the midline (Figure 3). Initially, a “closed” Sylvian fissure is observed, with both frontoparietal and temporal opercula juxtaposed. As we approach the midline, the insular gyri can be seen in the depth of the lateral sulcus. The parasagittal sections selected for the observation display a “closed” lateral sulcus and a visible inferior frontal sulcus, navigating the slices from lateral to medial until the last one before the insula appears, given that some accessory sulci are usually more superficial and do not reach the insula (Sprung-Much and Petrides, 2018). We chose parasagittal sections rather than surface observation to navigate the intra-sulcal anatomy as some accessory sulci and rami do not always reach the brain surface therefore the full extent of intra-sulcal anatomy cannot be appreciated from the brain surface only (Foundas et al., 1998; Keller et al., 2007, 2009). Brains with absent rami of the lateral sulcus and double inferior precentral sulci were excluded (*n* = 6).

We used Chi-squared tests to evaluate whether the presence of the triangular and diagonal sulci depended on the hemisphere and whether this differed according to sex. The presence of significant interhemispheric differences in the number of sulci in the frontal and parietal operculum (i.e., anterior and posterior subcentral sulci and parietal notches) was assessed by a Wilcoxon paired test. To evaluate any potential allometric effect between brain size and the presence of accessory sulci, we correlated total intracranial volume (as a measure of brain size) with the number of accessory sulci found in each brain, i.e., diagonal and triangular sulci, and parietal notches, using Spearman’s rank correlation. Finally, to test whether brains with accessory sulci in the frontal operculum have more folds in the parietal operculum, we applied Pearson’s Chi-squared Test. All tests were performed in Rstudio (v 3.6.3)^3^.

### Morphometric analysis

MR images were processed using Freesurfer (v 6.0.0.0)^4^, a combination of tools that divides the cortex into parcels for each hemisphere and quantifies gray matter volume. The processing was carried out using the automated and validated pipeline “recon-all” due to its wide use in structural brain studies and its verified accuracy, reliability, and validity (Fischl et al., 1999; Fischl, 2012). “Recon-all” includes preprocessing steps: e.g., removal of non-brain tissue, an automatic Talairach transformation (although the registration of the volume and surfaces happens as part of the process, the recon-all output used here is in the subject’s native space), segmentation of the subcortical white and deep gray matter volumetric structures, intensity normalization, tessellation of the gray-white matter boundary, an automatic topology correction, and the location of the gray/white matter and gray matter/cerebrospinal fluid borders where significant shifts in intensity define the transition to the other tissue class, among others. Then, the algorithm converts the volume into a surface through multiple deformation procedures that aim to register the image on a pre-existing atlas of the cerebral cortex that matches cortical geometry across subjects. Finally, the algorithm allowed the cortex parcellation in each hemisphere into sulcal and gyral regions. To this end, “recon-all” implements a probabilistic atlas as a reference. In this case, we used the atlas by Destrieux et al. (2010) and Fischl et al. (1999). The automatic parcellation of each brain was visually evaluated and possible errors were manually corrected. Among the results of the “recon-all”, we obtained the cortical volume (in mm3) of cortical gyri and sulci of the left and right FPO in the native space of each subject. As a measure of brain size, we used the estimated total intracranial volume (ICV) from Freesurfer (using mri_segstats; Buckner et al., 2004).

To assess the effect of accessory sulci on the gray matter content of gyri and sulci of the FPO, we applied linear models, where the accessory sulci were introduced as independent variables and the volume of cortical regions as the dependent variables. The scanner site of each sample was also included as a covariate to account for any effects related to the acquisition of the MRI sequences (Jovicich et al., 2009), as well as the putative differences between the two samples. Resulting *p*-values were adjusted through a False Discovery Rate test (FDR), to minimize type 1 error rate (Benjamini and Yekutieli, 2001). Corrected *p*-values < 0.05 were considered significant. Volume values were standardized by the estimated total intracranial volume of each subject. Again, all tests were performed in Rstudio (v 3.6.3)^3^.

## Results

### Intra and interindividual variability in the presence of accessory sulci

The triangular sulcus was found in 51% of the individuals in our sample (Table 2). Though more frequently found in the right hemisphere (Table 2), the presence of this sulcus was not significantly different between hemispheres, χ^2^_(1, *N* = 100)_ = 0.17, *p* > 0.05. The diagonal sulcus was present in 60% of the individuals and was more frequently found in the left than in the right hemisphere or bilaterally. However, these interhemispheric differences were not significant χ^2^_(1, *N* = 100)_ = 0.33, *p* > 0.05. In the frontal and parietal operculum (i.e., anterior and posterior subcentral sulci and parietal notches), we found accessory sulci in the precentral, postcentral, and supramarginal gyri. These sulci extend from the lateral sulcus in variable numbers ranging from 1 to 7 (Table 2), and with notable leftward asymmetry. The number of these accessory sulci in the frontal and parietal operculum was significantly higher in the left than in the right hemisphere (Wilcoxon test, *p* < 0.01). The presence of accessory sulci was not statistically different between sexes (diagonal sulcus: χ^2^_(1, *N* = 100)_ = 0.30, *p* > 0.05; triangular sulcus: χ^2^_(1, *N* = 100)_ = 0.22, *p* > 0.05; parietal notches: χ^2^_(1, *N* = 100)_ = 0.27, *p* > 0.05).

Regarding the relationship between brain size and the folding in the FPO, the correlation was very low and non-significant (*R* = 0.0048; *p* > 0.05), indicating that larger brains do not necessarily have more folds (or more accessory sulci). In addition, brains with accessory sulci in the frontal operculum do not necessarily have more folds in the parietal operculum (triangular sulcus: χ^2^_(6, *N* = 200)_ = 4.56, *p* > 0.05; diagonal sulcus: χ^2^_(6, *N* = 200)_ = 5.76, *p* > 0.05).

**Table 2:** Percentage of hemispheres with accessory sulci.

	ds	trs	pn (3)	pn (4)	pn (5)	pn (6)	pn (7)
RH	19	24	32	16	8	1	0
LH	23	14	17	20	17	4	1
Both	18	13	9	14	3	0	0

*Abbreviations: RH, right hemisphere; LH, left hemisphere; ds, diagonal sulcus; trs: triangular sulcus; pn, parietal notches. The number between parentheses indicates the number of accessory sulci in the parietal operculum (even the anterior and posterior subcentral sulci)*.

### Accessory sulci and gray matter content

The results of the linear models showed that the presence of the diagonal sulcus had a significant effect on the cortical volume of the inferior frontal sulcus (Table 3) and on the cortical volume of the pars opercularis in the inferior frontal gyrus (*p* < 0.05, Table 3). Hemispheres with a diagonal sulcus exhibited a significantly larger relative volume of pars opercularis and inferior frontal sulcus (*M* = 0.0024, SD = 0.00036; *M* = 0.0022, SD = 0.00039, respectively) compared to those without accessory sulci (*M* = 0.0022, SD = 0.00032; *M* = 0.0021, SD = 0.00037, respectively). The joint presence of the diagonal and triangular sulci showed a significant effect on the volume of the parcel of the horizontal ramus of the lateral sulcus (Table 3). The interaction between sex and the presence of accessory sulci did not display a significant effect after the FDR adjustment.

**Table 3:** Influence of the presence of frontal operculum accessory sulci on the volume of parcels of the region.

Parcels	trs (*p*-values)	ds (*p*-values)	trs^*^ds (*p*-values)
Inferior frontal sulcus	0.611	**0.030**	0.460
Pars opercularis (IFg)	0.651	**0.030**	0.765
Pars triangularis (IFg)	0.960	0.792	0.733
Horizontal ramus of the lateral sulcus	0.319	0.173	**0.021**
Ascending anterior ramus of the lateral sulcus	0.960	0.561	0.460
Posterior ramus of the lateral sulcus	0.133	0.792	0.460

*P-values in bold were significant after FDR. The asterisk indicates that analysis was performed considering the potential interaction of the presence of both accessory sulci on the volume of each parcel in the same hemisphere. Abbreviations: ds, diagonal sulcus (when diagonal sulcus was present, regardless if triangular sulcus was present); IFg, inferior frontal gyrus; trs, triangular sulcus (when triangular sulcus was present, regardless if diagonal sulcus was present)*.

The number of parietal notches and subcentral sulci showed a significant effect on the volume of the parcel of the angular gyrus (Table 4). The interaction between the presence of these accessory sulci in the frontal and parietal operculum and sex was not statistically significant.

**Table 4:** Influence of the presence of parietal operculum accessory and subcentral sulci on the volume of parcels of the region.

Parcels	pn (*p*-values)
Subcentral gyrus and sulci	0.469
Angular gyrus	**0.000**
Supramarginal gyrus	0.929
Postcentral gyrus	0.270
Precentral gyrus	0.106
Horizontal ramus of the lateral sulcus	0.066
Ascending anterior ramus of the lateral sulcus	0.832
Posterior ramus of the lateral sulcus	0.106

*P-values in bold were significant after FDR. Abbreviations: pn, parietal notches (plus the subcentral sulci)*.

## Discussion

In this study, we evaluated the anatomical and morphometric variability of the FPO. For this aim, we combined a visual survey of the presence of accessory sulci and outcomes of the gyri and sulci quantification. An interesting finding of this study was the high prevalence of accessory sulci in the FPO, particularly those located in the parietal operculum, which has not been reported before. The triangular and diagonal sulci were found in more than half of the subjects, and accessory sulci of the parietal operculum were also frequent. Finally, we found that these accessory sulci influence gray matter volume.

### Intra and inter-individual variability in accessory sulci

The sulcal variation in the frontal operculum is not exclusive to humans, as is shown by the presence of the diagonal sulcus, a bifurcated or incomplete inferior frontal sulcus, and an inferior precentral sulcus in non-human primates (e.g., Sherwood et al., 2003). Although these structures are not necessarily homologous across species and might differ in their cytoarchitectonic structure (Falk, 2014), their presence indicates that secondary or tertiary folds are common at least in hominids (Hathaway et al., 2022). The high prevalence of diagonal and triangular sulci found here is consistent with studies performed on *ex vivo* brains in smaller samples (Tomaiuolo et al., 1999; Keller et al., 2007, 2009; Idowu et al., 2014; Eser Ocak and Kocaelı, 2017). Our results show that the diagonal and triangular sulci are mostly found in one hemisphere (in 42% and 38% of subjects, respectively) rather than bilaterally (18% and 13%, respectively), i.e., the expression of these accessory sulci is mainly asymmetric. Similar to previous studies (Idowu et al., 2014; Eser Ocak and Kocaelı, 2017), the triangular sulcus was found indistinctly in one or the other hemisphere. A lack of directional asymmetry also characterized the diagonal sulcus, in agreement with the findings reported by Sprung-Much and Petrides (2018) in a sample of MRI volumes. This result contrasts with other MRI studies that found a leftward or rightward prevalence, although such results might be biased due to the small sample size analyzed in these studies (i.e., less than 50 individuals; Idowu et al., 2014; Eser Ocak and Kocaelı, 2017). Regarding the subcentral and parietal operculum, a recent study examined the morphological and functional variability of two sulci placed on both sides of the central sulcus, the anterior and posterior subcentral sulci (Eichert et al., 2021). Here, in addition to these subcentral sulci, we described a set of sulci with high prevalence and intra- and inter-individual variability ranging from 1 to 5 in each hemisphere. Interestingly, we observed that the subcentral sulci and the parietal notches are deeper than the diagonal and triangular sulci (Figure 3) suggesting that they might develop earlier during ontogeny, given that later developing folds are shallower (Lohmann et al., 1999). Further studies evaluating the prevalence and shape changes of the sulci analyzed here throughout ontogeny are needed for a better understanding of the brain folding process. In this sense, the study performed here can be expanded using other tools, such as the BrainVISA software, that identify sulci in individual cortical surfaces preserving the inter-individual variability of secondary and tertiary sulci (Rivière et al., 2003). Finally, we showed a lack of association between the presence of accessory sulci and sex, which is in line with previous results (e.g., Idowu et al., 2014; Eser Ocak and Kocaelı, 2017).

### Effect of accessory sulci on gray matter quantification

We expected a significant effect of the anatomical variants on the cortical measurements according to the location of the accessory sulci. Our results show that the diagonal sulcus influenced the estimations of gray matter volume of the inferior frontal sulcus and pars opercularis. Such influence could be explained by the criteria applied in the automatic parcellation. According to the atlas’ authors, the “inconstant” or “variable” sulci of the frontal operculum, i.e., diagonal and triangular sulci, are included within the inferior frontal sulcus (Destrieux et al., 2010), even though these sulci are not considered an anatomical extension of the inferior frontal sulcus (Petrides and Pandya, 2004).

Regarding the parietal notches, the authors of the atlas used in the present work do not specify in which parcels these were included. These sulci seem to be short rami from the lateral sulcus, so are expected to be included in the corresponding parcel. However, the sulci identified were included within parcels such as pars opercularis, insula, and supramarginal gyrus, rather than the lateral sulcus. Also, these parietal notches influenced the volume of a parcel anatomically located more distant from the lateral sulcus: the angular gyrus. One possible explanation is that the volume of this parcel may be affected by the anatomic boundary demarcation (Seghier, 2013). In this sense, the automatic parcellation of the supramarginal gyrus used here (Destrieux et al., 2010) defines a virtual line from the tip of the posterior subcentral sulcus (“temporo-parietal line”) as one of the boundaries. Thus, a variable number of accessory sulci in the subcentral and supramarginal gyri could affect the location of this virtual line from subject to subject and affect the inferior parietal lobule parcel’s demarcation. Also, the higher number of parietal notches in the left hemisphere could be related to a reduction of the posterior region of the lateral sulcus, resulting in the smaller volume of the left angular gyrus found in previous studies (e.g., Chiarello et al., 2016). In sum, our results suggest that accessory sulci can have an effect on the automatic parcellation and quantification of parcels that do not match with their anatomical location. In this regard, the approach followed here, which labels gyri and sulci based on the registration to an atlas previously defined, could be complemented with approaches based on individual fold detection (e.g., those implemented in BrainVISA or Mars Atlas), given that they allow the identification and quantification of secondary and even tertiary sulci with high inter-individual variability (Jiang et al., 2021).

A potential shortcoming of this investigation is the lack of left-handed and ambidextrous subjects. The analysis of larger samples with left-handed and ambidextrous volunteers with a wider age range is necessary to explore in more detail the variable expression of accessory sulci in healthy individuals and the effect of these anatomical variants on automatic parcellations. Further studies are also required to better assess whether the high prevalence of accessory sulci found here is a particular characteristic of the population under study, taking into account the differences in brain structure found among populations (Zilles et al., 2001; Xu et al., 2017; Tang et al., 2018; Kang et al., 2020). Previous studies evaluated the presence of accessory sulci in individuals sampled from western populations (Lou et al., 2020), with a genetic and environmental background different from the South American sample included here. This bias in MRI databases is widely recognized, and more efforts are needed to increase the number of samples from underrepresented populations (Falk et al., 2013; Sirugo et al., 2019; Bethlehem et al., 2022). Additionally, the functional correlates of the accessory sulci are still underexplored and deserve more attention. Recent studies report significant associations between the depth or the length of tertiary or accessory sulci in the prefrontal cortex and the functional and cognitive domains in predictions regarding reasoning skills in children and hallucinations in patients with schizophrenia (Garrison et al., 2015; Voorhies et al., 2021; Miller and Weiner, 2022). These findings highlight the relevance of studying the accessory sulci in the human brain.

## Conclusions

A notable finding of this study was the high frequency of anatomical variants described as “accessory sulci”, which in the sample analyzed appear to be more common than what was assumed by the brain atlases used for automatic parcellation. The accessory sulci in the FPO showed a significant influence on the gray matter volume of some parcels, which has implications for quantitative imaging, a growing field within modern radiology and neuroscience. Moreover, these accessory sulci could be a source of variation among individuals, and they could affect the direction of asymmetry in the FPO when present. For this reason, the presence of accessory sulci should be considered in future studies as a significant source of variation in automatic quantifications of cortical volume. Moreover, it would probably be interesting to review how variable these types of sulci are and use a manual labeling system, as used in this study, to train automated labeling algorithms to identify such sulci.

## Data Availability Statement

The original contributions presented in the study are included in the article/Supplementary-material, further inquiries can be directed to the corresponding author/s.

## Ethics Statement

The studies involving human participants were reviewed and approved by Ethical Committees of the Hospital El Cruce and Hospital Angel Roffo, Buenos Aires, Argentina. The patients/participants provided their written informed consent to participate in this study.

## Author Contributions

MV-A conceived and designed the study, collected the data, and performed the analysis. LA-F, AB, JP, and MM contributed to data collection. MB conceived and designed the study (neuroanatomist), and drafted the manuscript. PG conceived and designed the study, and drafted the manuscript. All authors contributed to the article and approved the submitted version.

## Footnotes

^1^ https://www.nitrc.org/projects/dcm2nii/

^2^ https://www.slicer.org/

^3^ https://rstudio.com/

^4^ http://surfer.nmr.mgh.harvard.edu
